# Immobilization of Wnt Fragment Peptides on Magnetic Nanoparticles or Synthetic Surfaces Regulate Wnt Signaling Kinetics

**DOI:** 10.3390/ijms231710164

**Published:** 2022-09-05

**Authors:** Bin Hu, Michael Rotherham, Neil Farrow, Paul Roach, Jon Dobson, Alicia J. El Haj

**Affiliations:** 1 School of Pharmacy and Bioengineering, Guy Hilton Research Center, Keele University, Thornburrow Drive, Hartshill, Stoke-on-Trent, Staffordshire ST4 7QB, UK; 2 CAS Key Laboratory for Biomedical Effects of Nanomaterials and Nanosafety & CAS Center for Excellence in Nanoscience, National Center for Nanoscience and Technology, Beijing 100190, China; 3 Healthcare Technologies Institute, School of Chemical Engineering, University of Birmingham, Heritage Building, Mindelsohn Way, Birmingham B15 2TH, UK; 4 Department of Chemistry, Loughborough University, Leicestershire, Loughborough LE11 3TU, UK; 5 J Crayton Pruitt Family Department of Biomedical Engineering, University of Florida, Gainesville, FL 32611, USA

**Keywords:** magnetic nanoparticle, magnetic force bioreactor, nanomagnetic actuation, peptide immobilization, Wnt/β-catenin, mechanotransduction, tissue engineering

## Abstract

Wnt signaling plays an important role in embryogenesis and adult stem cell homeostasis. Its diminished activation is implicated in osteoporosis and degenerative neural diseases. However, systematic administration of Wnt-signaling agonists carries risk, as aberrantly activated Wnt/β-catenin signaling is linked to cancer. Therefore, technologies for local modulation and control of Wnt signaling targeted to specific sites of disease or degeneration have potential therapeutic value in the treatment of degenerative diseases. We reported a facile approach to locally activate the canonical Wnt signaling cascade using nanomagnetic actuation or ligand immobilized platforms. Using a human embryonic kidney (HEK293) Luc-TCF/LEF reporter cell line, we demonstrated that targeting the cell membrane Wnt receptor, Frizzled 2, with peptide-tagged magnetic nanoparticles (MNPs) triggered canonical Wnt signaling transduction when exposed to a high-gradient, time-varying magnetic field, and the induced TCF/LEF signal transduction was shown to be avidity-dependent. We also demonstrated that the peptide retained signaling activity after functionalization onto glass surfaces, providing a versatile platform for drug discovery or recreation of the cell niche. In conclusion, these results showed that peptide-mediated Wnt signaling kinetics depended not only on ligand concentration but also on the presentation method of the ligand, which may be further modulated by magnetic actuation. This has important implications when designing future therapeutic platforms involving Wnt mimetics.

## 1. Introduction

Wnt signaling plays a crucial role in a variety of biological processes, including embryogenesis and stem cell maintenance in adult mammals. Dysregulation of Wnt signaling causes a wide spectrum of diseases, including cancer, bone disorders and neurodegeneration [1]. The binding of a canonical Wnt to a Frizzled receptor and a co-receptor of the low-density lipoprotein receptor-related protein family (LRP5/6) on the cell membrane results in the formation of a ternary signaling module that dissembles the ‘destruction complex’ (casein kinase I (CK1), glycogen synthase kinase 3β (GSK3β), axin, and adenomatous polyposis coli gene product (APC)). Consequently, β-catenin accumulates in the cytoplasm and eventually translocates to the nucleus, where it combines with members of the T cell factor/lymphoid enhancer factor (TCF/LEF) to modulate the transcription of Wnt-responsive genes [1,2]. Similar to many signal transduction pathways, Wnt ligand binding induces receptor dimerization that triggers a conformational change of the receptors, which is relayed by phosphorylation of key target proteins [3].

Enhancing tissue repair by activating endogenous stem cells holds promise for a variety of disorders. However, the progress of exploiting Wnt signaling for regenerative medicine has been hindered by difficulties in producing sufficient quantities of recombinant Wnts for systemic administration [4]. Moreover, the potential risks of off-target activation of this pathway associated with systemic administration cannot be ignored. Therefore, the development of techniques and platforms for the localized, spatial targeting and modulation of Wnt signaling may provide a suitable therapeutic option.

Mechanical forces are also critical factors that regulate embryogenesis and tissue remodeling and are increasingly being exploited for tissue engineering applications [5,6,7]. In vivo and in vitro studies have revealed that Wnt and β-catenin signaling is also responsive to mechanical loading [8,9,10]. At the nanoscale, the application of pico-newton (pN) forces can induce protein interactions and direct lineage engagement [11,12]. Nanomagnetic actuation can be used to remotely apply these highly localized mechanical forces, in the pico-newton and nano-newton (nN) range, directly to membrane mechano-receptors mediated by functionalized micro- or nano-particles (MNP) [13,14]. This approach allows a degree of spatial and temporal control over local cellular signaling [15]. Our group has previously used a magnetic force bioreactor consisting of oscillating arrays of permanent magnets providing high field gradients and flux densities up to 250 mT. This system has been combined with MNP to remotely activate a range of cell receptors and ion channels to control cell fate and harness for engineering cells and tissues at the nanoscale [16,17,18,19]. The size of the MNP in these studies (~250 nm) was carefully selected to balance the trade-off between biocompatibility, particle size (and subsequent force generation) and the magnetic susceptibility of the material.

The spatial and temporal presentation of Wnts are important factors in determining stem cell fate; the immobilization of Wnt proteins onto substrates provides an attractive and controllable way of directing cell behavior [20]. Walker et al. used MNP coated with a temperature-sensitive polymer to entrap Wnt proteins [21]. Magnetic heating of the MNP caused the release of Wnt from the polymer matrix and induced Mesenchymal stem cell proliferation. Another study showed that Wnt immobilized on beads can provide a platform to induce asymmetric cell division that produces divergent cell fates in daughter cells. Habib et al. used Wnt-immobilized beads to induce asymmetric cell division in embryonic stem cells [22]. After cell division, the bead proximal to the Wnt-bead maintained stem cell marker expressions while the cell distal to the Wnt-bead expressed differentiation markers. It was also possible to create an immobilized Wnt platform that recapitulates Wnt signaling gradients that are a feature of stem cell niches in vivo. Directional presentation of Wnt to stem cells in this manner differentially regulated mesenchymal stem cell fate [23,24,25].

In the present study, a Wnt fragment peptide (UM206) was used to target MNP to the cell surface Frizzled receptors. UM206 is a peptide based on a conserved sequence of Wnta3a/Wnt5a developed by Blankesteijn et al. The peptide exhibited different signaling activity depending on its conformation and the presence of a disulfide bridge between cysteine residues within the peptide. UM206 in a linear conformation (UM206_L) was shown to be inactive, while in a cyclic, disulfide bridged conformation (UM206_C) displayed agonistic properties in Frizzled over-expressing HEK cells [26]. In our previous studies, we found the activity of either conformation of UM206 to vary according to the target cell type and whether the peptide was applied in a free state (soluble form) or conjugated to MNP [27,28].

In this study, we demonstrated that nanomagnetic actuation of Wnt fragment peptide (UM206)-functionalized MNPs could locally modulate the canonical Wnt signaling cascade in vitro, and the induced TCF/LEF signal transduction was shown to be avidity-dependent. We also showed that Wnt fragment peptides could be immobilized on synthetic surfaces and retained some signaling capacity.

## 2. Results

### 2.1. HEK293 Reporter Cell Line Expresses Target Frizzled Receptor

**Figure 1:**
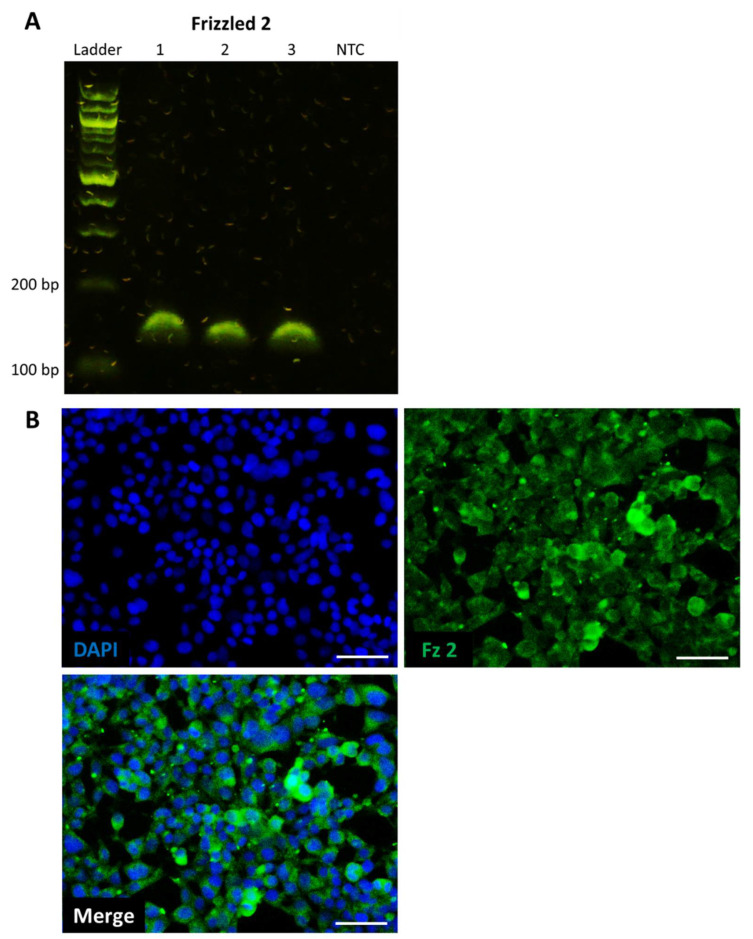
Frizzled 2 is expressed by HEK293 reporter cells. (**A**) Reverse transcription-PCR confirmed Frizzled 2 expression at transcriptional level, *n* = 3 (1–3 = sample number, NTC = no template control). (**B**) Immunocytochemistry confirmed Frizzled 2 expression at translational level, representative images of *n* = 3; Frizzled 2 is shown in green (**top left**), and cell nuclei are shown in blue by DAPI stain (**top right**), merge channel is shown below, scale bar represents 50 μm.

We first confirmed that the HEK293 TCF/LEF reporter cell line expressed the main target receptor for UM206, Frizzled 2. PCR and immunocytochemistry both confirmed that HEK293 expressed the Frizzled 2 receptor at both the transcriptional and translational levels, respectively (Figure 1A,B).

### 2.2. Soluble Peptides Have No Overall Effect on TCF/LEF Signal Transduction in Reporter Cell Line

**Figure 2:**
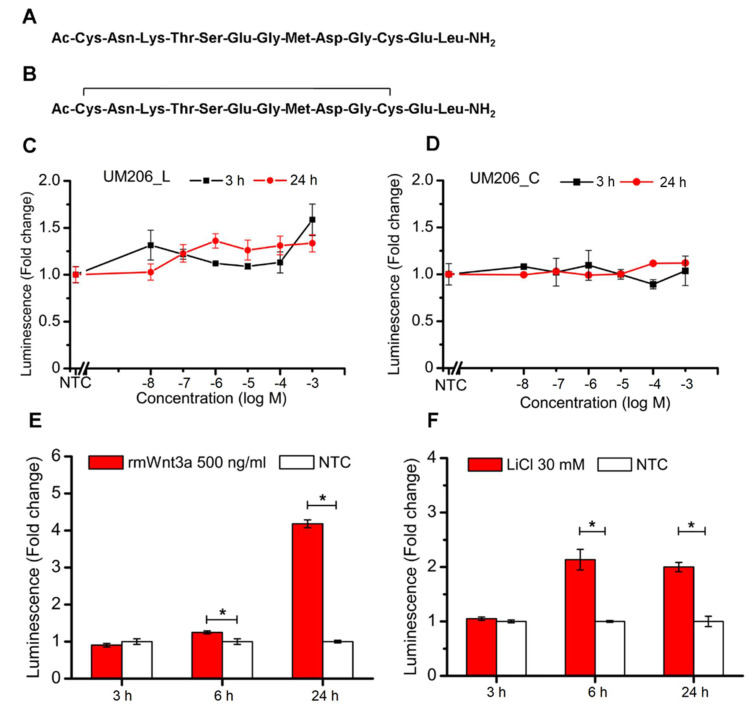
Effect of peptides and two agonists on the activation of canonical Wnt signaling transduction. (**A**,**B**) Sequence of UM206_L, UM206_C. (**C**,**D**) TCF/LEF signaling transduction induced by indicated peptide concentration for UM206_L (**C**), UM206_C (**D**) 3 h (■) and 24 h (●) post-treatment in HEK293 Luc-TCF/LEF cells (*n* = 3). (**E**,**F**) The stimulatory effect of 500 ng/mL rmWnt3a (**E**) and 30 mM LiCl (**F**) treatment on TCF/LEF transduction at the indicated time points (*n* = 4). In (**C**–**F**), luminescence was presented as fold change with respect to non-treated control (NTC), which was set to 1. The data are expressed as mean ± SEM obtained from two independent experiments (* *p* < 0.05).

We then tested the ability of soluble UM206_L and UM206_C (Figure 2A,B) to activate the Wnt/β-catenin signaling using a luciferase reporter transcriptionally controlled by a TCF/LEF promotor. When the peptides were applied directly to cells, neither UM206_L nor UM206_C peptides showed stable inhibitory or stimulatory effects on TCF/LEF signaling during the duration of the experiment (Figure 2C,D). In contrast, the application of rmWnt3a boosted TCF/LEF signal transduction by 0.24 (±0.04)- and 3.18 (±0.11)-fold at 6 h and 24 h post-treatment, respectively (Figure 2E). Another canonical Wnt agonist, LiCl treatment, enhanced TCF/LEF signal transduction 1.14 (±0.19)- and 1.00 (±0.09)-fold at 6 h and 24 h post-treatment, respectively (Figure 2F). It was consistently observed that the onset of enhanced TCF/LEF signal transduction occurred later than 3 h post-treatment in HEK293 Luc-LEF/TCF cells in response to either rmWnt3a or LiCl treatment.

### 2.3. Characterization of Magnetic Nanoparticle (MNP)

We next explored whether the immobilization of the peptide onto the surface of MNP altered its signaling efficacy. We used carbodiimide activation to form a peptide bond between MNP and the UM206 peptide. We then assessed whether combining these peptide-functionalized MNPs with mechanical stimulation of Frizzled provided by magnetic fields was capable of initiating Wnt/β-catenin signaling. We characterized the peptide-functionalized MNP using dynamic light scattering (DLS) and zeta potential to assess changes in particle size and surface charge after peptide coating (Table 1). Results showed a clear shift in surface potential upon peptide conjugation, reflecting the change in surface potential that resulted from the deposition of peptides onto the surface of the MNP. However, there was a less obvious size shift after peptide conjugation. This was broadly consistent with our previous results [27] and may be due to the small peptides used for conjugation and the existence of a hydration layer on the MNPs in the solution. We also characterized the MNP by TEM and FT-IR. TEM imaging showed that the cluster morphology of the MNP was unaffected by coating with UM206 (Supplementary Figure S1). The FT-IR spectra of the MNP showed an absorbance peak at ~1660 cm^−1^, corresponding to amide I C = O stretch, which was most prominent in the coated MNP groups. A peak of ~1500 cm^−1^ was also observed, corresponding to amide II N-H bending and was more prominent in the UM206_C coated groups. A smaller peak was also observed around ~3000 cm^−1^ in the coated MNP groups, corresponding to the C-H stretch (Supplementary Figure S2). We next used a protein assay to determine the amount of peptide bound to the MNP after coating with different concentrations. The peptide concentration on the MNP surface was slightly increased when coated with 10 μg of UM206_L (0.37 μg/mL) compared to 1 μg of UM206_L (0.10 μg/mL). Coating with UM206_C resulted in overall more peptides on the MNP surface compared to UM206_L, but a similar number of peptides was observed after coating with either 1 μg of UM206_C (0.62 μg/mL) or with 10 μg of UM206_C (0.59 μg/mL).

### 2.4. Nanomechanical Activation of Functionalized MNPs Stimulates β-Catenin Mediated TCF/LEF Signal Transduction

**Figure 3:**
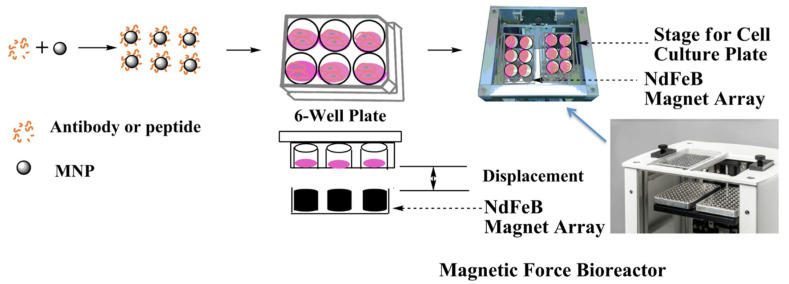
Schematics of the nanomagnetic actuation technique using a magnetic force bioreactor (MFB). MNPs are first functionalized with antibodies or peptides and then incubated with cells. After removing unbound MNPs, cells are exposed to a high-gradient, time-varying magnetic field generated by vertically oscillating NdFeB magnet arrays mounted on stepper motors under computer control [17].

We then assessed Wnt signal transduction using the HEK293 Luc-TCF/LEF luciferase reporter cells in response to UM206 functionalized MNP and high-gradient 1 Hz magnetic fields provided by the magnetic force bioreactor (Figure 3).

**Figure 4:**
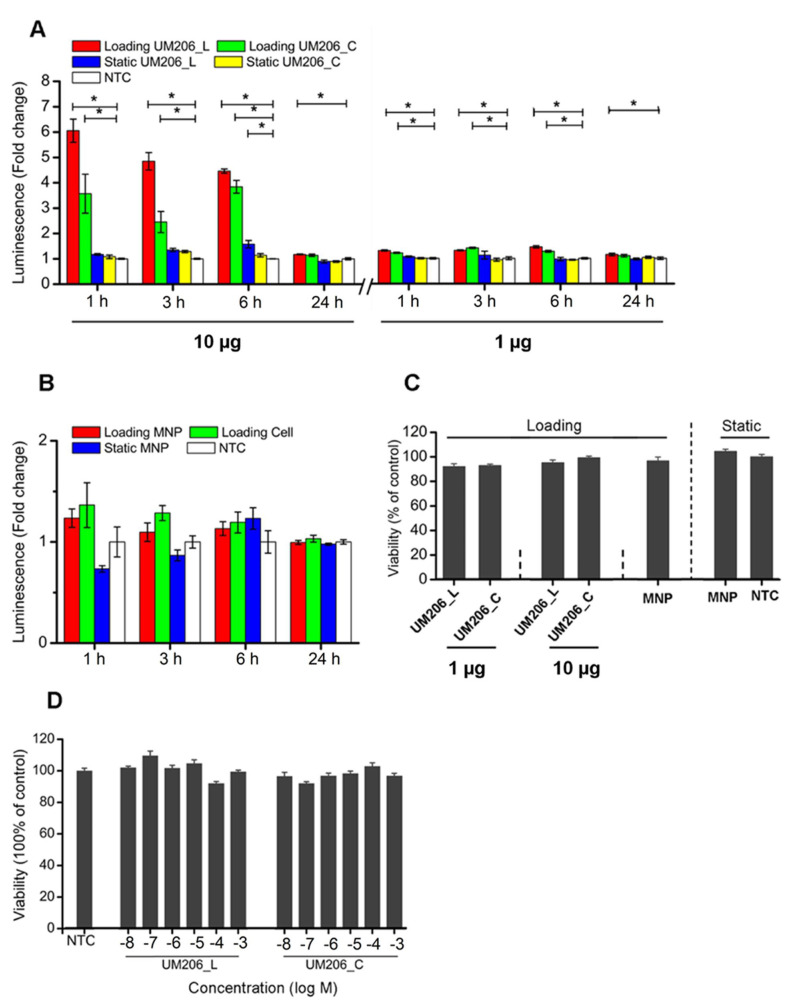
Dose response of UM206 functionalized MNPs on TCF/LEF signaling induction in HEK293 Luc-TCF/LEF cells. (**A**) Left and right: the starting ratio of peptide-to-MNP is 10 μg-per-mg MNP and 1 μg-per-mg MNP, respectively. (**B**) Effects of control MNPs without conjugation. (**C**) Viability of HEK293 cells in response to various treatments and (**D**) to indicated concentration of peptides. “Loading” indicates MFB exposure, and “Static” indicates no exposure to MFBs. In (**A**,**B**), luminescence was defined as fold change with respect to the non-treated control (NTC) group. For (**A**–**D**), data are presented as mean ± SEM, *n* = 4 and shown here is a representative set of results of two independent experiments (* *p* < 0.05).

UM206_L-conjugated MNPs exposed to the MFB (Loading UM206_L) activated TCF/LEF signaling in HEK293 Luc-TCF/LEF cells (Figure 4A). TCF/LEF transduction increased by 5.05 ± 0.46, 2.72 ± 0.26, 3.46 ± 0.85 and 0.17 ± 0.02-fold at 1, 3, 6 and 24 h post-initiation of magnetic stimulation, respectively. UM206_C-conjugated MNPs combined with MFB exposure (Loading UM206_C) also activated TCF/LEF signaling at all monitored time points but with less potency. The fold increases of signal transduction were 2.57 ± 0.77, 0.88 ± 0.32 and 2.84 ± 0.25 at 1, 3 and 6 h, respectively, and the stimulatory effect leveled off at 24 h. UM206_L-conjugated MNP incubation without MFB exposure also showed a stimulatory effect at 6 h, with a 1.58 ± 0.15-fold change. We also assessed canonical Wnt pathway activation by immunostaining for the active form of the messenger protein β-catenin in response to UM206 functionalized MNP. Both UM206_C and UM206_L (either 1 μg or 10 μg coating) induced a low level of nuclear localization of active β-catenin over the base levels observed in the non-treated and unconjugated-MNP control groups. The effect was more pronounced after 1 h of MFB exposure, with noticeable increases in nuclear-active β-catenin over the controls after treatment with either UM206_C or UM206_L (either 1 μg or 10 μg coating). The level of nuclear-active β-catenin in UM206-MNP groups was comparable to the Wnt3a control group after 24 h (Supplementary Figure S3).

Given that the dense population of biomolecules conjugated to the MNP surface may cause steric hindrance, a lower starting peptide quantity (1 μg peptide-per-mg MNP) for MNP conjugation was also investigated. Results revealed that the lower-density peptides appeared to attenuate the potency of the stimulatory effect of peptide conjugation combined with MFB exposure in HEK293 Luc-TCF/LEF cells. As is shown in Figure 4A, with the lower starting peptide concentration, UM206_L-conjugated MNPs combined with MFB exposure (Loading UM206_C) induced TCF/LEF signal transduction in HEK293 cells and the signal intensity fold changes were 1.31 ± 0.03, 1.31 ± 0.02, 1.45 ± 0.05 and 1.15 ± 0.05 folds at 1, 3, 6 and 24 h post-initiation of magnetic stimulation respectively. UM206_C-conjugated MNPs combined with MFB exposure (Loading UM206_C) also promoted TCF/LEF signaling at 1, 3 and 6 h but with less efficacy compared with Loading UM206_L, and the stimulatory effect diminished at 24 h. The fold changes of signal transduction were 1.21 ± 0.02, 1.41 ± 0.03 and 1.27 ± 0.04, at 1, 3 and 6 h, respectively, compared with NTC.

This suggested that with a starting quantity of 10μg peptide-per-mg MNP, the density of conjugated peptide molecules on the surfaces of the MNPs retained peptide bioactivity and was not sufficient to cause steric hindrance. Control groups treated with non-conjugated-MNPs or magnetic fields alone showed no statistically significant enhancement in TCF/LEF signal transduction following exposure (Figure 4B).

### 2.5. Magnetic Stimulation Has No Influence on Cell Viability

We also investigated whether the biocompatibility of the MNP was affected by UM206 functionalization. We used an MTT assay to assess mitochondrial activity and cell proliferation in the tested cells in response to the MNP. Results revealed that peptide-conjugated MNPs combined with MFB exposure neither compromised short-term cell viability nor induced proliferation when analyzed 24 h post-treatment (Figure 4C). Free-peptide treatment at the μM level was also tested for comparison, and no inhibitory effects were observed (Figure 4D).

### 2.6. Immobilised UM206 Retains Activity on Glass Substrate

**Figure 5:**
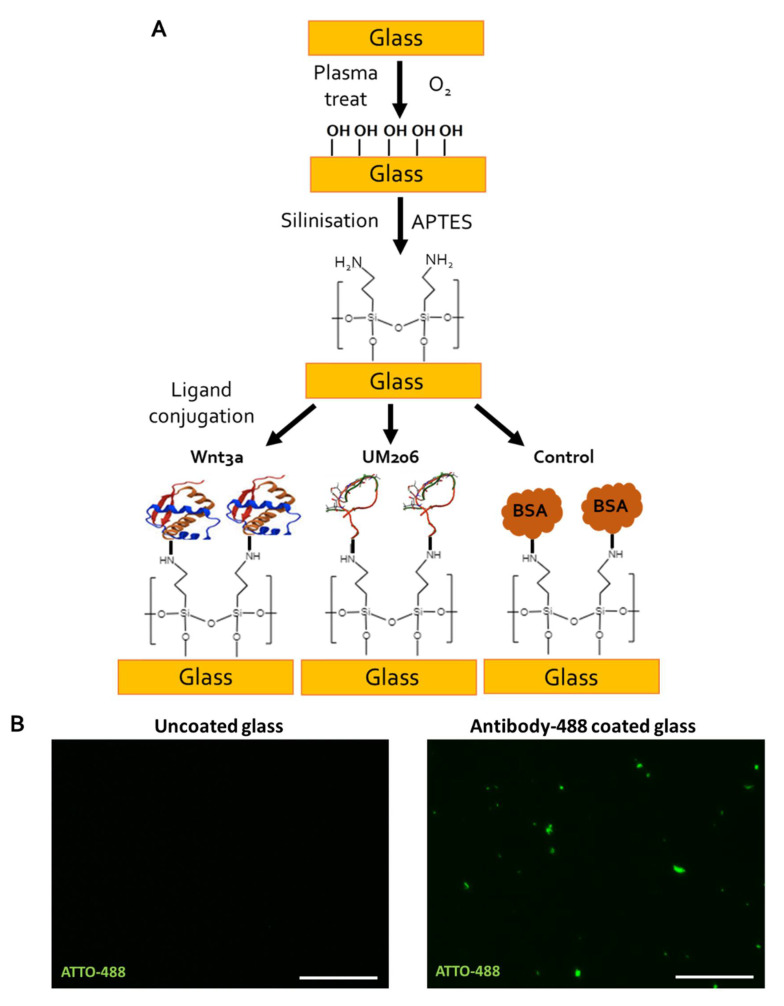

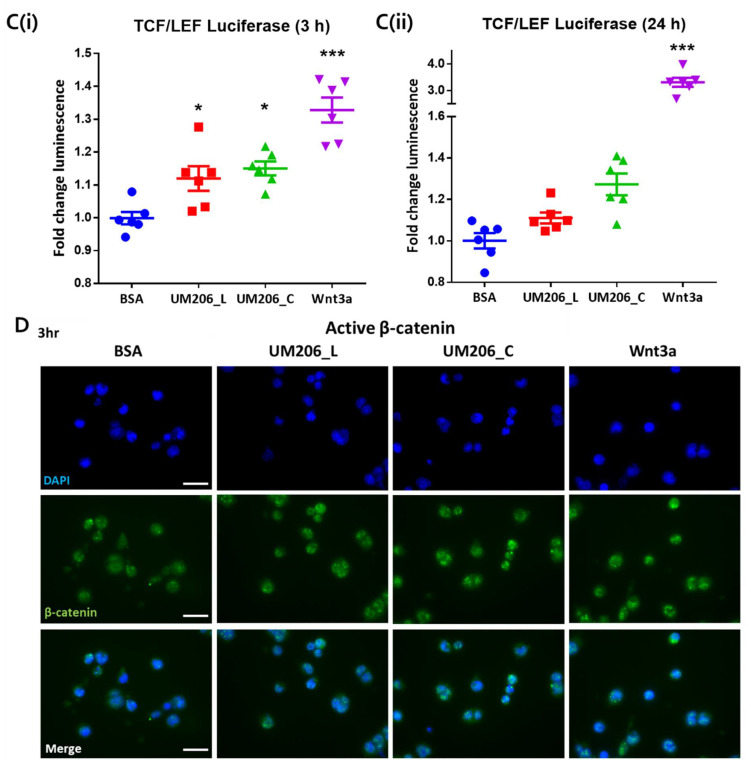
Immobilized UM206 peptide retained mild signaling activity on glass substrate. (**A**) Schematic of UM206 immobilization process onto amine-functionalized glass coverslips using oxygen plasma activation, APTES treatment, then ligand conjugation. (**B**) Fluorescent images of uncoated glass (**left**) and glass substrate coated with antibody-ATTO-488 (**right**) confirmed protein immobilization on glass surface. Bar represents 50 µm, images representative of *n* = 6. (**C**) HEK293 Luc-TCF/LEF reporter was mildly activated by both immobilized UM206_L and UM206_C to a comparable level as immobilized Wnt3a after 3 h (**C**(**i**)) but remained at similar levels after 24 h (**C**(**ii**)). Data are presented as mean fold change ± SEM, *n* = 6. Shown here are representative results of two independent experiments (* *p* < 0.05, *** *p* < 0.001). (**D**) Immunofluorescent images of active β-catenin in response to UM206 functionalized surfaces. A base level of activated β-catenin was observed in the control (BSA) coated group. An increase in activated nuclear β-catenin was observed in response to surfaces coated with UM206_C, UM206_L, or with Wnt3a positive control after 3 h. Representative images of *n* = 3 shown. Scale bar represents 50 μm.

Our studies above demonstrated how nanoparticle targeting of Wnt receptors on the surface of a cell could lead to activation. However, these properties may change when peptides are bound to a substrate with cells grown on top in a monolayer. To define the responses of peptide ligands bound onto a substrate, we also examined the use of UM206 as a Wnt-stimulating platform by immobilizing the peptide on glass coverslips. Oxygen plasma treatment and APTES were first used to create an amine-functionalized glass surface to promote protein binding (Figure 5A), following a method described by Okuchi et al. [25]. We confirmed the protein binding properties of the activated surface by conjugating a fluorescent antibody to the surface. Fluorescent microscopy confirmed antibody-ATTO-488 immobilization on the surface, while no fluorescence was detected on uncoated glass (Figure 5B). UM206 peptides were then conjugated to glass coverslips to form an immobilized peptide presenting platform onto which HEK293 Luc-TCF/LEF luciferase reporter cells were seeded. Both immobilized UM206_L and UM206_C induced mild short-term increases in TCF/LEF signal transduction after 3 h, which was comparable to the luciferase signal induced by immobilized Wnt3a (Figure 5C(i)). After 24 h, signal transduction, induced by UM206 peptides, remained similar to that at 3 h, while immobilized Wnt3a induced a threefold increase in signal transduction (Figure 5C(ii)). Increased mobilization of active β-catenin over the BSA-coated control group was also observed in response to UM206_C, UM206_L and the Wnt3a coated surfaces after 3h (Figure 5D).

## 3. Discussion

This study outlined facile and enabling technologies to remotely control the canonical Wnt/β-catenin signaling pathway. Our study demonstrates that the bioactivity of Wnt mimetics using the Wnt fragment peptide UM206 is dependent on the presentation method to the target cells. Wnt pathway activity significantly varied depending on whether UM206 was presented to cells in soluble form. This resulted in inconsistent activity, immobilized on synthetic glass surfaces, which provided weak agonistic activity or was conjugated to magnetic nanoparticles which displayed more robust signaling activation. This suggested that the activation mechanism was concentration, conformation or spatially dependent. All these factors were dependent on the chemical method and peptide concentrations used during immobilization and were, therefore important considerations in studies examining the activity of tethered proteins and peptides. Although this study is not the first one published in this area, it is the first mechanistic study to be performed and provided an interesting and important insight into the effect that the peptide presentation method had on downstream Wnt signaling activity. The current study also provided a solid foundation for other published works in this area involving magnetic activation of Wnt, which focused on in vitro signaling in human mesenchymal stem cells (hMSCs), neuronal differentiation of SH-SY5Y and an ex vivo bone tissue engineering model using hMSCs injected into chicken femurs respectively [27,28,29]. Recent work has also shown that the nanomagnetic activation approach and immobilized platforms are promising tools that are readily translatable as platforms for drug discovery or stem cell niche recreation, or for tissue engineering applications [23,25,30]. Our current study supported these works and demonstrated the biocompatibility and adaptable nature of the two approaches.

Our group has previously focused on the use of antibodies for MNP targeting, including for targeting Wnt signaling [29]. Indeed, other groups have also demonstrated the efficacy of activating the Wnt pathway in this manner using tetravalent antibodies [31]. Having said this, there remains the potential for steric hindrance when using concentrated immobilized antibodies and antibody immunoreactivity may be altered during the MNP conjugation reaction. These factors could impact membrane receptor binding efficiency or affect receptor conformation changes induced by magnetic stimulation. These potential pitfalls remain important to consider when developing these approaches for future applications. To circumvent these issues, we applied functional Wnt-fragment peptides to mediate Frizzled binding. The activity of UM206 has been shown by our group and others to not only be conformationally dependent but also varied according to the target cell and species [26,27,28]. UM206_L and UM206_C were initially reported to antagonize and agonize Wnt signaling, respectively, in Frizzled 2 overexpressing-HEK293 cells but not in wild-type HEK293 cells [26]. In the current study, when the peptide was functionalized to MNPs and combined with exposure to a time-varying, high-gradient magnetic field or immobilized on glass surfaces, both conformations of the peptide displayed agonistic signaling activity in cells without overexpression of Frizzled 2. The effect on signaling was found to be more dependent on the ligand concentration used during MNP coating rather than the presence or absence of the disulfide bridge in the peptide, which displayed signaling activity under both conformations. Interestingly, our results showed that increasing the UM206 concentration during coating ten-fold only marginally increased the amount of UM206_L ligands bound to the MNP surface (and did not affect the amount of UM206_C on the MNP), but this still had a clear effect on signaling activity. We also observed overall more UM206_C coating on the MNP surface despite this conformation showing slightly less efficacy than UM206_L. This suggested that ligand immobilization, steric effects, and peptide concentration during MNP functionalization were the most important factors that determined whether enough ligands were provided in the correct orientation for Frizzled binding and induction of signaling activity, at least in cells with endogenous Frizzled 2 expression. The onset of the TCF/LEF signaling transduction triggered by nanomagnetic actuation was also earlier than that induced by Wnt3a or LiCl treatment, suggesting that this approach promoted relatively fast but short-lived signaling propagation through peptide-MNPs, which may be temporarily enhanced further with mechanotransduction.

The underlying mechanisms for nanomagnetic actuation are not completely understood [32]. However, a growing body of literature reveals that mechanical stimulation can induce functional conformation transitions in mechano-responsive proteins, which alter binding properties and enzymatic functions [33,34]. Recent work by Schihada et al. has suggested that a conformational change in the transmembrane domains of Frizzled in response to Wnt binding may be part of the receptor activation mechanism in HEK293 [35]. In the context of the present study, it was, therefore, conceivable that the forces exerted by the MFBs might be changing the conformation of Frizzled 2 in a similar manner, and this initiated diverse responses to UM206_L- and UM206_C-functionalized MNPs in the HEK293 cells.

It is also known that mechanical stimuli are capable of modulating endocytosis. In this sense, when cell membrane tension increases after force application, endocytosis may be slowed down [36]. The result of this would be an increase in the availability of Frizzled and other co-receptors at the cell membrane for Wnt signaling propagation. Moreover, the peptide-functionalized MNPs used in these experiments could also have acted as complex scaffolds to facilitate receptor clustering and interaction of multiple components of the Wnt/β-catenin signaling pathway by stabilizing the extra- or intracellular complex. In support of this hypothesis, recent work from our group showed that magnetic actuation of Frizzled 2 in hMSC by UM206-MNP may induce a degree of Frizzled receptor clustering at the cell membrane [27].

## 4. Materials and Methods

### 4.1. Magnetic Force Bioreactor

The magnetic force bioreactor (MFB) (MICA Biosystems, Ltd., Solihull, UK) consists of horizontal arrays of cylindrical NdFeB magnets aligned to the wells of each multi-well plate. The magnet arrays move vertically and the frequency and amplitude of the oscillations of the array are controlled. The field strength produced by the magnet array in the vicinity of the cells is in the region of ≤120 mT with a field gradient of 3.3–11.0 Tm^−1^, resulting in forces of approximately 2 pN per MNP [37].

### 4.2. Construction of a Stably Transfected Reporter Cell Line

Luciferase plasmid expression vector pcDNA3.1 (Gaussian Luciferase) was kindly donated by Alexander Faussner (Ludwig-Maximilians-Universität, Germany), and its expression was controlled by TCF/LEF elements [38]. Competent cells were purchased from Sigma (Gillingham, UK). Plasmid constructs were amplified in bacterial cells using standard techniques. Isolation and purification of plasmid DNA were performed using the EndoFree Plasmid Maxi Kit (Qiagen, Manchester, UK) according to the manufacturer’s instructions. The HEK293 cell line was obtained from the American Type Culture Collection (ATCC, Middlesex, UK). HEK293 cells stably expressing the 7 × TCF/LEF-luciferase plasmid were transfected using Lipofectamine 2000 (Invitrogen, Paisley, UK) and screened by culturing in DMEM medium containing 10% FBS, 1% nonessential amino acids and 500 μg/mL G418 (PAA, Yeovil, Somerset, UK). Several transfection conditions were tested. The HEK293 TCF/LEF-GLuc cells with the highest signal-to-background ratio after challenging with rmWnt3a (Biotechne, Abingdon, UK) were grown at 37 °C in a 5% CO_2_ 95% humidified air incubator in DMEM, containing 10% FBS, 1% nonessential amino acids, 500 μg/mL G418.

### 4.3. Polymerase Chain Reaction

Total RNA was extracted using an RNA extraction kit (Bioline, London, UK) according to the manufacturer’s instructions. Reverse transcription was performed on 1 μg RNA using a high-capacity reverse transcription kit (Applied Biosystems, Warrington, UK). PCR reaction mixes were prepared using diluted cDNA mixed with PCR master mix (Applied Biosystems, Warrington, UK) and commercially available primers for Frizzled 2 (Qiagen, Manchester, UK). Thermocycling was performed on an AriaMx qPCR system (Agilent, Stockport, UK). PCR products were resolved on a 2% agarose gel and imaged using an E-gel powersnap electrophoresis device (Invitrogen, Paisley, UK).

### 4.4. Immunocytochemistry

For the establishment of Frizzled receptor expression, cells were fixed with 4% PFA in PBS (Fisher, Loughborough, UK) for 15 min, permeabilized with 0.1% Triton-X in PBS for 10 min, washed with PBS, then blocked with 2% BSA/PBS for 2 h. Cells were then stained with anti-Frizzled 2 (Invitrogen, Paisley, UK) diluted 1:50 in 1% BSA/PBS and incubated overnight at 4 °C. After washing with PBS, cells were stained with chicken anti-goat-488 (Invitrogen, Paisley, UK) diluted 1:2000 in 1% BSA/PBS and incubated for 2 h at room temperature. Cells were washed with PBS, then counterstained with DAPI (Sigma, Gillingham, UK) for 15 min and stored in PBS.

For assessment of active β-catenin mobilization, media were aspirated and cells fixed with 90% methanol (Fisher, Loughborough, UK) for 10 min after treatment. Cells were permeabilized with 0.1% Triton-X in PBS (Sigma Gillingham, UK) for 10 min, then blocked in 2% BSA (Fisher, Loughborough, UK) in PBS for 1 h at room temp. Cells were then incubated overnight at 4 °C with anti-active β-catenin antibodies (Millipore, Watford, UK) diluted 1:1000 in 1% BSA in PBS. Cells were washed in PBS, then incubated with anti-mouse-FITC secondary antibodies (Sigma, Gillingham, UK) diluted 1:1000 in 1% BSA in PBS for 1 h at room temp. Cells were then washed with PBS and counterstained with DAPI (Sigma Gillingham, UK). Fluorescence microscopy was performed on an EVOS M5000 microscope (Invitrogen, Paisley, UK).

### 4.5. Effect of Synthetic Peptides on TCF/LEF Signaling Transduction

UM206 (UM206_L) and UM206 (S-S) (referred to as UM206_C) were synthesized by Pepceuticals (Enderby, UK) according to the sequences shown in Figure 2A,B. The purity of each of the peptides was not less than 75%. HEK293 Luc-LEF/TCF cells were plated onto 96-well plates at a density of 80,000 cells per well. After an overnight culture, a fresh medium, inclusive of various concentrations of peptides at a 10^−8^–10^−3^ M level, was administered to the cells. The cells without peptide treatment were used as the control, and the cells receiving the 500 ng/mL rmWnt3a treatment were used as the positive control. The TCF/LEF signaling transduction was measured at 3 and 24 h post-treatment by assaying the luciferase activity using a Gaussia Luciferase Flash Assay Kit (ThermoScientific, Wilmington, DE, USA) according to the manufacturer’s protocol. The luminescence was detected using a Synergy 2 (Biotek, Potton, UK) spectrometer.

### 4.6. Preparation of Peptide-Conjugated Magnetic Nanoparticles

MNP consisted of 250 nm cluster-typed superparamagnetic dextran-iron oxide composite nanoparticles (Micromod, Rostock, Germany) with surface carboxyl functional groups and a saturation magnetization of >63 emu/g iron. The amine groups present in the peptide were conjugated to the surface carboxyl groups of the MNP according to the manufacturer’s protocol. 12 mg of 1-ethyl-3-(3-dimethylaminopropyl)-carbodiimide hydrochloride (EDAC) and 24 mg of N-hydroxysuccinimide (NHS) were dissolved in 2 mL of 0.5M 2-(4-morpholino)ethanesulphonic acid (MES) buffer and 40 μL of this solution was added to 0.2 mL of MNP suspension (10 mg/mL). The suspension was incubated with continuous mixing for 1 h at room temperature to activate the MNPs, after which the particles were washed with 0.1 M MES buffer and resuspended in 0.2 mL 0.1 M MES buffer containing 1 or 10 μg of the peptide. After 3 h of continuous mixing at room temperature, 20 μL of 25 mM glycine in PBS buffer was added to the suspension and incubated for 30 min with continuous mixing. The resulting particle suspension was washed and resuspended in 0.2 mL PBS buffer containing 0.1% BSA (Bioreagent grade, >98% purity, Fisher, Loughborough, UK) and stored at 4 °C.

### 4.7. Characterisation of Magnetic Nanoparticles

#### 4.7.1. Zetasizing and Zeta Potential

The size and surface charge of MNPs with or without peptide conjugation were measured using a ZetaSizer (Malvern, Worcestershire, UK) at 25 °C when dispersed in PBS or distilled H_2_O.

#### 4.7.2. Total Protein Assay

The amount of peptide on the MNP surface after coating was assessed using a NanoOrange assay (Invitrogen, Paisley, UK). Coated MNP solutions were diluted 10× in a working NanoOrange reagent prepared according to the manufacturer’s instructions. Samples were heated to 95 °C for 10 min, cooled to room temperature for 20 min and briefly centrifuged. The fluorescence intensity of the samples was measured with an excitation wavelength of 470 nm and emission at 570 nm. Protein concentration on the MNP was calculated using a BSA standard curve.

#### 4.7.3. Transmission Electron Microscopy

MNP was diluted 1:10 in distilled water to a concentration of 0.1 mg/mL, 2 μL of MNP were pipetted onto Formvar and carbon film copper mesh grids (Agar Scientific, London, UK; S162H). The grids were air dried and then imaged on a JEM-100CX II transmission electron microscope (JEOL, Peabody, MA, USA) operating at 100 kV. Electron micrographs were captured using a Megaview III digital camera using Radius software (EMSIS GmbH, Münster, Germany).

#### 4.7.4. Fourier-Transform Infrared Spectroscopy (FTIR)

MNP samples at a concentration of 1 mg/mL were drop-dried and powder-applied to an IN10mx FTIR (ThermoFisher, Loughborough, UK) fitted with a Ge ATR; the system was purged continuously with dry air during analysis. All spectra were recorded at room temperature, with 256 scans being averaged at a resolution of 4 cm^−1^.

### 4.8. Magneto-Mechanical Stimulation of HEK293 Luc-LEF/TCF Cells

HEK293 Luc-LEF/TCF cells were plated onto six-well plates at a density of 1.5 × 10^6^ cells per well. After 24 h of culture, the serum was withdrawn from the culture medium for 1 h, cells were incubated with 25 μg of MNP (approx. 1.3 × 10^3^ MNP per cell) either with or without peptide conjugation. After 30 min of incubation, cells were washed with PBS to remove unbound MNPs, replaced with a fresh DMEM medium containing 2% FBS and 1% non-essential amino acid, and then put into the MFB for a set time period, with the frequency set at 1 Hz. Cells without the MNP addition and MFB exposure were employed as controls. The medium was collected at predetermined time points and analyzed using a Gaussia Luciferase Flash Assay Kit (ThermoScientific, Loughborough, UK) per the manufacturer’s instructions.

### 4.9. Cell Viability

HEK293 Luc-LEF/TCF cells were seeded onto 96-well plates at the density of 80,000 cells per well. After overnight culture, cells were treated as described in the previous section. All samples were analyzed 24 h post-initiation of magnetic stimulation. The starting ratio of peptide to MNP was 10 μg peptide per mg of MNP for conjugation.

After various treatments, 10 μL of the MTT solution (5 mg/mL; Dojindo, Huntingdon, UK) was added to each well and incubated for 4 h at 37 °C. After removing the medium, 200 µL of DMSO was added to each well to dissolve the produced solids, aided by gentle shaking. The absorbance was measured using a Synergy 2 spectrometer (450 nm), and readings were normalized to that of the non-treated control group (set as 100%).

### 4.10. Functionalization of Glass Coverslips

Circular glass coverslips (10 mm in diameter, SLS, Nottingham, UK) were functionalized using a protocol based on work by Okuchi et al. [25]. First, coverslips were oxygen plasma treated for 2 min using a Piezobrush PZ2 handheld plasma device (Relyon Plasma, Regensburg, Germany) operating under a 2 psi O_2_ gas flow. Coverslips were then amine-functionalized using alternating solutions of acidified 2% APTES (Sigma Gillingham, UK) in 90% EtOH (pH 4.0) for 30 min followed by 2% APTES in 90% EtOH (pH 12.0) for 30 min, which was repeated twice. Coverslips were then washed in 90% EtOH, transferred to 24-well plates then rinsed in PBS and air dried for 1 h before addition of either 1 µg of UM206_L, UM206_C peptide or 100 ng Wnt3a (Biotechne, Abingdon, UK) dissolved in 20 µL of PBS. Control surfaces were coated in 20 µL of 1% BSA/PBS (Fisher) or 1 µg anti-Mouse-ATTO-488 (Sigma, Gillingham, UK). Coverslips were left to coat overnight at 4 °C. Coating solutions were then aspirated. The coverslips were washed with PBS and then incubated with the culture media for 30 min to block unreacted groups; 1.10^5^ cells were then seeded onto the functionalized coverslips in 20 µL and left to attach for 1 h before topping up the wells with 500 µL media.

### 4.11. Statistics

Data are presented as the mean ± standard error of the mean (SEM). Differences between groups were examined for statistical significance with a one-way analysis of variance (ANOVA) using the Tukey least-significance test for post-hoc comparisons. A minimal probability value of less than 5% was considered a statistically significant difference.

## 5. Conclusions

This study demonstrated how the application of peptide-functionalized MNPs, combined with exposure to a cyclic magnetic field via an MFB, or the immobilization of Wnt fragment peptides to synthetic surfaces, could modulate Wnt signaling. Moreover, the use of peptide-conjugated MNPs or immobilized Wnt-fragment surfaces displayed a highly specific and temporal targeting potential. These strategies potentially could be used as local signaling modulators or growth factor platforms with applications in drug discovery, cell niche modeling or the treatment of disease-damaged tissues in tissue engineering and regenerative medicine. Crucially, such strategies could avoid the widespread activation of Wnt signaling in the biosystem, thereby preventing potential side effects.

## Figures and Tables

**Table 1:** Size and surface potential of MNP with or without peptide conjugation.

		Size (nm)	Zeta (mV)
Control	MNP-Uncoated	314.7 ± 3.3	−30.8 ± 0.4
10 μg peptide per 1 mg particle	MNP-UM206_L	319.0 ± 0.7	−16.8 ± 0.7
	MNP-UM206_C	329.3 ± 4.7	−14.9 ± 0.2
1 μg peptide per 1 mg particle	MNP-UM206_L	323.0 ± 2.1	−15.2 ± 0.3
	MNP-UM206_C	317.9 ± 2.9	−14.2 ± 0.1

## Data Availability

The data presented in this study are available in the article or Supplementary Materials.

## Supplementary Materials

The following supporting information can be downloaded at: https://www.mdpi.com/article/10.3390/ijms231710164/s1.
