# Supplementary material for: Immobilization of Wnt Fragment Peptides on Magnetic Nanoparticles or Synthetic Surfaces Regulate Wnt Signaling Kinetics
Source: Int J Mol Sci. 2022 Sep 5;23(17):10164. doi: 10.3390/ijms231710164 (PMC9456016; doi:10.3390/ijms231710164)
Supplement: Supplementary file 1 [file ijms-23-10164-s001.zip › ijms-1715358-supplementary.pdf]

Supplementary material

Immobilization of Wnt Fragment Peptides on Magnetic Nanoparticles or Synthetic Surfaces Regulate Wnt Signaling Kinetics

Bin Hu [1,2,†], Michael Rotherham [3,*,†], Neil Farrow [1], Paul Roach [1,4], Jon Dobson [5,‡] and Alicia J. El Haj [1,3,*,‡]

[1] School of Pharmacy and Bioengineering, Keele University, Guy Hilton Research Center, Thornburrow Drive, Hartshill, Stoke-on-trent, Staffordshire, ST4 7QB, UK

[2] CAS Key Laboratory for Biomedical Effects of Nanomaterials and Nanosafety & CAS Center for Excellence in Nanoscience, National Center for Nanoscience and Technology, Beijing 100190, China

[3] Healthcare Technologies Institute, School of Chemical Engineering, Heritage Building, Mindelsohn Way, University of Birmingham, Birmingham, B15 2TH, UK.

[4] Department of Chemistry, Loughborough University, Loughborough, Leicestershire, LE11 3TU, UK

[5] J Crayton Pruitt Family Department of Biomedical Engineering, University of Florida, Gainesville, Florida 32611, USA

* Correspondence: m.rotherham@bham.ac.uk (M.R.); A.ElHaj@bham.ac.uk (A.J.E.H.); Tel. (A.J.E.H.): +44 0121-371-8001

† B.H. and M.R. are joint first authors

‡ J.D. and A.J.E.H are joint senior authors

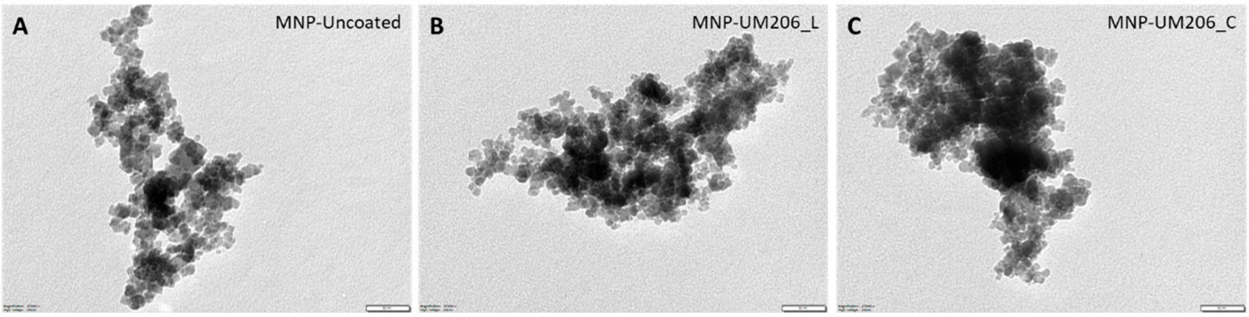

Supplementary Figure S1: Transmission electron micrographs of Blank-MNP (MNP-Uncoated) (a), 10µg-Linear-UM206 coated MNP (MNP-UM206_L) (b) and 10µg-Cyclic-UM206 coated MNP (MNP-UM206_C) (c), magnification is 270k, bar represents 50 nm.

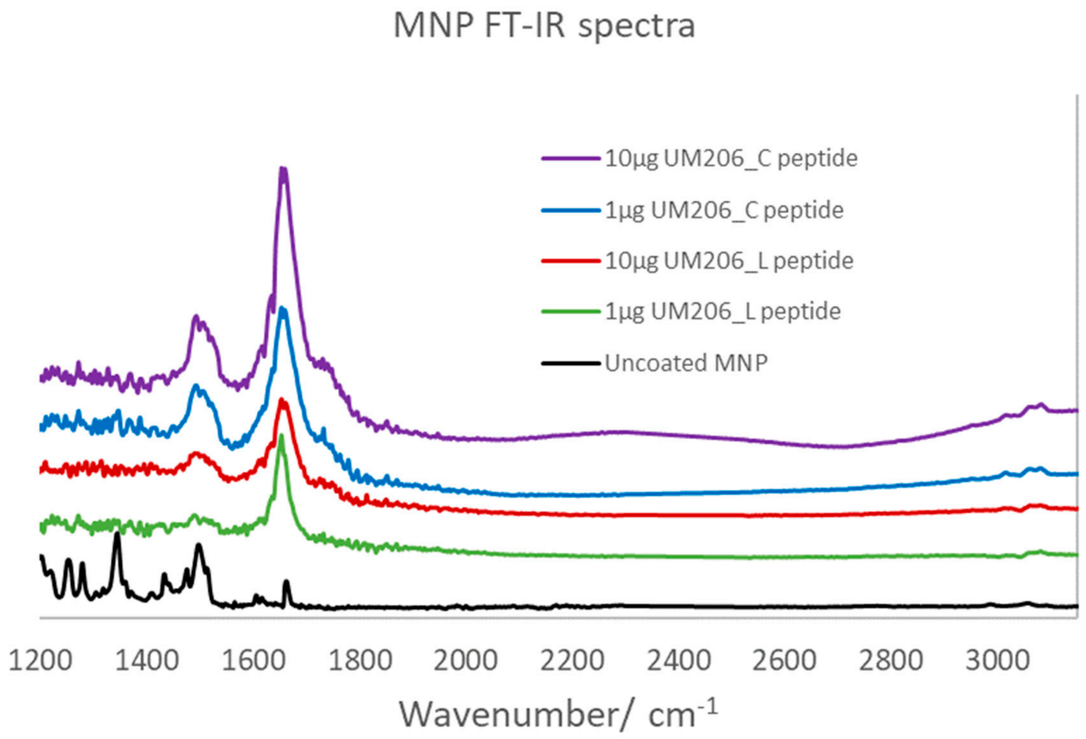

Supplementary Figure S2: FT-IR spectra of Control MNP (Uncoated MNP) (**Black**), 1µg-Linear-UM206 coated MNP (1µg UM206_L peptide) (Green), 10µg-Linear-UM206 coated MNP (10µg UM206_L peptide) (Red), 1µg-Cyclic-UM206 coated MNP (1µg UM206_C peptide) (Blue) and 10µg-Cyclic-UM206 coated MNP (10µg UM206_C peptide) (Purple). Absorbance peaks at ~1660cm^{-1}, corresponding to amide I C=O stretch, ~1500cm^{-1} corresponding to amide II N-H bending and a minor peak around ~3000cm^{-1}, corresponding to C-H stretch were all present, especially in the coated MNP groups. Spectra represent an average of 256 scans at a resolution of 4 cm^{-1}.

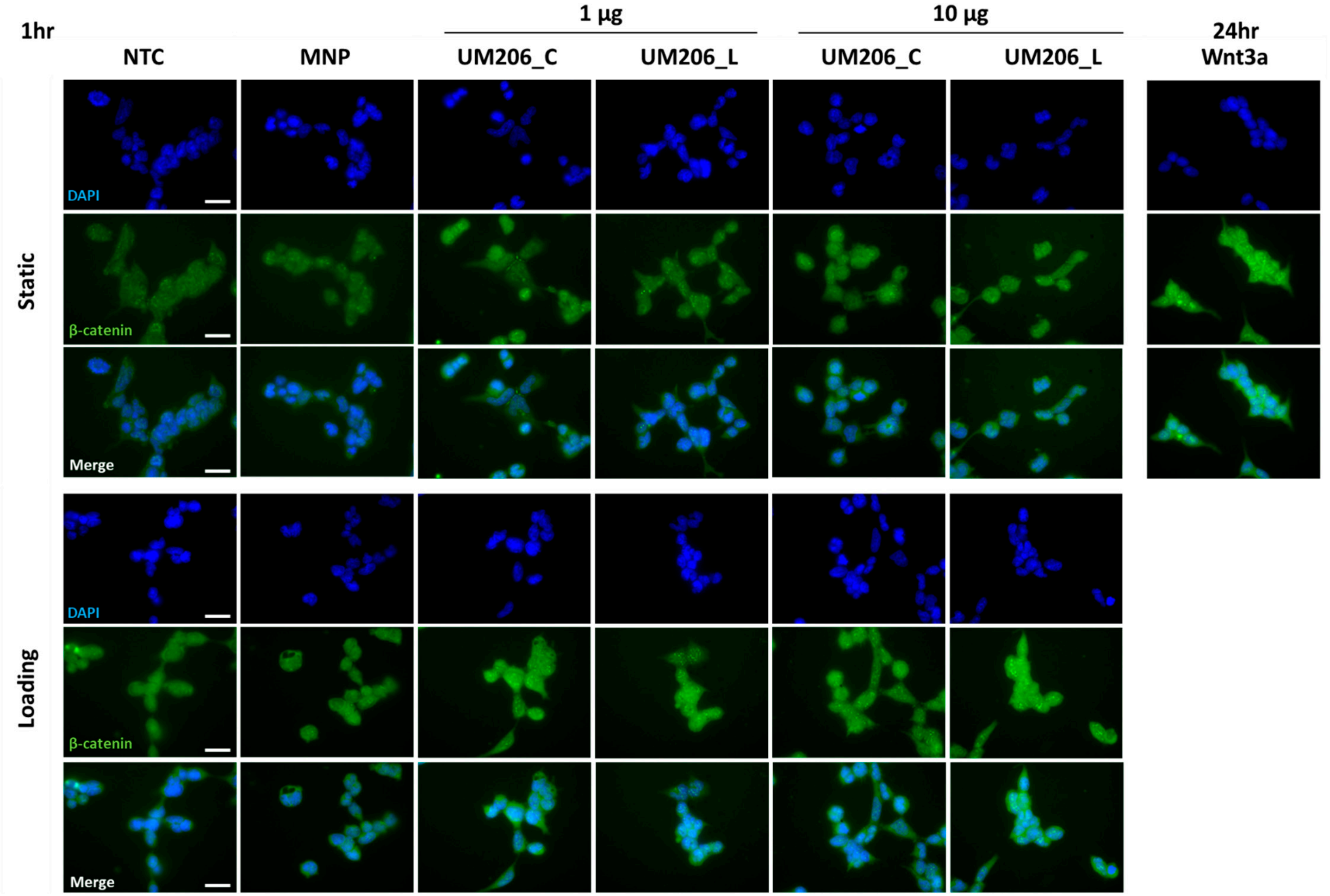

Supplementary Figure S3: Immunofluorescent images of active β-catenin in response to UM206 functionalised MNP after 1h treatment. A base level of active β-catenin was observed in the non-treated control (NTC) and uncoated MNP (MNP) with or without loading with MFB. A mild increase in active β-catenin was observed in response to static UM206_C and UM206_L with either 1 µg or 10 µg coating (top row). In contrast a clear increase in active β-catenin which was also mobilised to the nucleus was observed in response to the positive control Wnt3a after 24h. A similarly increased level of nuclear mobilised active β-catenin was also observed in cells treated with UM206_C and UM206_L with either 1 µg or 10 µg coating when loaded for 1h with MFB (bottom row). Representative images of n=3 shown. Scale bar represents 50 µm.
